# Alterations in Neuronal Nicotinic Acetylcholine Receptors in the Pathogenesis of Various Cognitive Impairments

**DOI:** 10.1111/cns.70069

**Published:** 2024-10-06

**Authors:** Zhi‐Zhong Guan

**Affiliations:** ^1^ Department of Pathology The Affiliated Hospital of Guizhou Medical University Guiyang P.R. China; ^2^ Key Laboratory of Endemic and Ethnic Diseases Guizhou Medical University, Ministry of Education and Provincial Key Laboratory of Medical Molecular Biology Guiyang P.R. China

**Keywords:** Alzheimer's disease, cognitive disorder, diabetes mellitus, nicotinic acetylcholine receptors, Parkinson's disease, schizophrenia, vascular dementia

## Abstract

Cognitive impairment is a typical symptom of both neurodegenerative and certain other diseases. In connection with these different pathologies, the etiology and neurological and metabolic changes associated with cognitive impairment must differ. Until these characteristics and differences are understood in greater detail, pharmacological treatment of the different forms of cognitive impairment remains suboptimal. Neurotransmitter receptors, including neuronal nicotinic acetylcholine receptors (nAChRs), dopamine receptors, and glutamine receptors, play key roles in the functions and metabolisms of the brain. Among these, the role of nAChRs in the development of cognitive impairment has attracted more and more attention. The present review summarizes what is presently known concerning the structure, distribution, metabolism, and function of nAChRs, as well as their involvement in major cognitive disorders such as Alzheimer's disease, Parkinson's disease, vascular dementia, schizophrenia, and diabetes mellitus. As will be discussed, the relevant scientific literature reveals clearly that the α4β2 and α7 nAChR subtypes and/or subunits of the receptors play major roles in maintaining cognitive function and in neuroprotection of the brain. Accordingly, focusing on these as targets of drug therapy can be expected to lead to breakthroughs in the treatment of cognitive disorders such as AD and schizophrenia.

## Graphical abstract

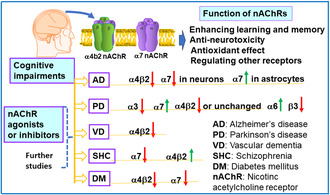

Changes of neuronal nAChRs in various cognitive impairments. The function of nAChRs is related to learning and memory and anti‐neurotoxicity. The modifications of nAChRs have their characteristics in different cognitive impairments. A variety of nAChR agonists and inhibitors have been and are being tested for treatment of cognitive impairment.

## 1 Introduction

Cognitive impairment is manifested primarily as memory loss, loss of the ability to use language, sensory insensitivity, and slowness of movements, among other symptoms. The degree of cognitive dysfunction, which varies widely, can be assessed by clinical and neuropsychological examination [1].

These symptoms are commonly associated with neurodegenerative diseases, such as Alzheimer's disease (AD), Parkinson's disease (PD), Creutzfeldt–Jakob disease, Huntington's disease, and amyotrophic lateral sclerosis [2], all of which are characterized by extensive deposits of misfolded protein aggregates, including β‐amyloid peptide (Aβ), hyperphosphorylated tau, α‐synuclein, Huntington protein, and TAR DNA‐binding protein 43 [3, 4]. At the same time, cognitive impairment is a feature of normal aging [5], as well as of other types of conditions [6], including vascular dementia (VD) [7], schizophrenia [8], type 2 diabetes mellitus (T2DM) [9], sleep disturbances [10], damage to the intestinal barrier [11], chronic fluorosis [12, 13, 14], and arsenic poisoning [15]. Moreover, there may even be a functional cause [16].

Research on the involvement of receptors for neurotransmitters in the development of cognitive impairment is expanding. Early evidence indicated a characteristic loss of neuronal nicotinic acetylcholine receptors (nAChRs) in brain tissues obtained in connection with autopsy of patients with AD, and the finding was subsequently confirmed in the observation in vivo by positron emission tomography (PET) [17]. Moreover, the reduction in the number of dopamine receptors associated with PD has been shown to be a consequence of the loss of dopaminergic nerve terminals [18]. Such a reduction is also associated with schizophrenia in both adolescents and adults and causes deficits in motivation, cognition, and sensory functions [19].

Furthermore, estrogen receptors and signaling improve cognition and neuroprotection via multiple neural systems, such as the dopaminergic, serotonergic, and glutamatergic systems, and disturbances in these processes are also associated with a variety of psychiatric disorders [20]. In addition, targeting metabotropic glutamate receptors can potentially provide a fundamentally new approach to symptomatic relief for patients with schizophrenia [21]. Agonists to the serotonin 2A receptor exhibit a potential to slow down or reverse brain atrophy, enhance cognitive function, and decelerate disease progression in patients with AD [22]. Moreover, the role of the α1‐adrenergic receptor in regulating synaptic efficacy and different types of memory indicates that this is a potentially valuable target in connection with treatment of a wide variety of neurological conditions associated with impaired cognition [23]. At the same time, emerging evidence suggests that targeting adenosine G protein‐coupled receptors prior to the development of clinical symptoms may mitigate accumulation of pathogenic Aβ and tau neurotoxicity, while improving cognition and memory [24].

Interestingly, among these receptors for neurotransmitters, neuronal nAChRs are involved in a variety of brain functions, including cognition, memory, and neuroprotection, and have consequently been investigated extensively in association with the development of numerous brain disorders [25, 26]. Therefore, the present review focuses on the role of the receptors in cognitive impairment, along with possible underlying molecular mechanisms.

## 2 Factors Involved in the Development of Cognitive Impairment

The different mechanisms underlying the development of neurodegenerative and other diseases associated with cognitive impairment involve many different factors, including modifications of genes, transformation of signaling pathways, alterations in protein molecules, and changes in metabolic procedures [27, 28, 29, 30]. AD, the most common neurodegenerative cause of dementia, is characterized by accumulation of extracellular neuritic plaques composed of Aβ and intracellular neurofibrillary tangles containing phosphorylated tau [29, 30]. In addition, apolipoprotein E, glycogen synthase kinase 3β, notch signaling pathway, and Wnt signaling pathway, among other factors, are also considered to play a role in the advancement of AD [27]. It has been proposed that perturbations in cellular energy metabolism, sensitivity to excitation or inhibition, and/or the release of neurotrophic factors overwhelm compensatory mechanisms, leading dysfunction of neuronal microcircuits and brain networks [28]. In the case of PD, the characteristic impairment of movement is accompanied by numerous other types of symptoms, including cognitive impairment which may occur at any stage of the disease [31]. In addition to the classic misfolding of nigrostriatal α‐synuclein and loss of dopaminergic neurons, a variety of mechanisms involving a number of other systems and peptides contribute to this neurodegeneration [31].

Vascular dementia (VD) or vascular cognitive impairment is due primarily to cerebrovascular injury [7]. The most common underlying mechanism is chronic age‐related dysregulation of cerebral blood flow, although additional factors such as inflammation and cardiovascular dysfunction also play a role [32].

Moreover, schizophrenia, which is not a typical neurodegenerative disease, is associated with obvious cognitive impairment [33]. The underlying mechanisms involve disturbances in neurochemical processes, including the functions of dopamine and of the glutamatergic N‐methyl‐D‐aspartate (NMDA) receptor, as well as changes in multiple genes [33].

In addition, both preclinical and epidemiological investigations have consistently revealed an association between diabetes mellitus and cognitive decline [34, 35]. In this case, the factors involved include hyperglycemia, dyslipidemia, hypertension, insulin resistance, inflammation, vascular disorder, systemic amylin dyshomeostasis, and calcium dysregulation [35]. In the case of subtle and more severe forms of cognitive dysfunction associated with diabetes mellitus, the mechanisms are likely to be multifactorial and differ [34].

## 3 The Structure, Metabolism, and Function of Neuronal nAChRs

**FIGURE 1:**
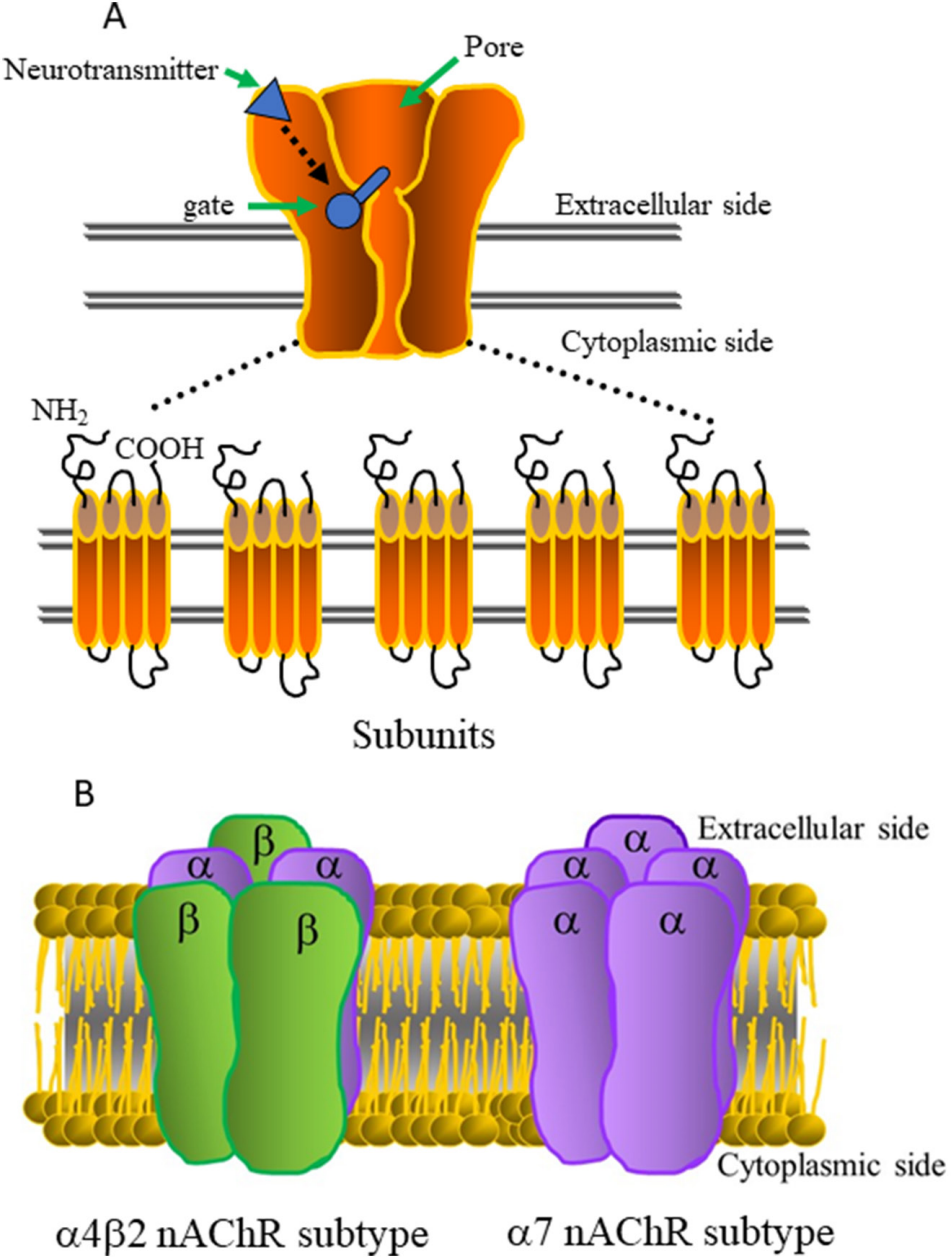
Structure of neuronal nAChRs. (A) Five subunits of nAChRs: each consists of four hydrophobic domains exposed to the cytoplasm with glycosylation sites, including transmembrane segments of the receptor, some of which are arranged on ion channels. Neurotransmitters produce their effects by action at one of a number of ligand‐binding sites that exist on the receptor‐ion channel complex. (B) Two major subtypes of neuronal nAChRs in brains: α4β2 and α7 nAChRs. The α4β2 subtypes assemble according to a general 2α3β stoichiometry, with the possibility of more than one α subunit within a pentamer. However, the α7 subtype is formed as functional homo‐oligomers composed of 5 (α7) subunits.

The nAChRs, the first neurotransmitter receptors to be identified [36], constitute a superfamily of homologous receptors, including cholinergic, gamma‐aminobutyric acid, glycine, serotonin, and glutamate receptors [37, 38]. In the brain, neuronal nAChRs are composed of two types of subunits, α and β, with genes encoding at least 9 α (α2–α10) and 3 β (β2–β4) subunits (Figure 1A) [39]. As in the case of the muscle type of nAChRs, the site that recognizes agonists of nAChRs is on the interface between α and β subunits [39].

Different combinations of α and β subunits produce functionally distinct subtypes of neuronal nAChRs in different regions of the brain (Figure 1B) [37, 39]. In general, each receptor contains 2α3β stoichiometry, with the possibility of more than one α subunit subtype within a pentamer [37]. However, α7, α8, and α9 subunits form functional homo‐oligomers consisting of five α subunits as a subtype [39].

The two most common nAChRs in the mammalian brain are the α4β2 heteromer and the α7 homomer [37], the former exhibiting high affinity for nicotine and the latter being the main site for binding α‐bungarotoxin. The α4β2 heteromeric receptor can contain two α4 and three β2 subunits or three α4 and two β2 subunits [40]. Both of these isoforms contain a pair of α4(+)/(−)β2 agonist‐binding sites and the other subunits neighboring these sites modify their contribution to nAChR activation [41]. In addition, each heteromeric neuronal nAChR contains two acetylcholine‐binding sites formed by pockets within the extracellular N‐terminal domain at the interface between adjacent subunits [42, 43, 44]. The situation is more complicated in the case of the homomeric α7 nAChR, where the interfaces between the subunits provide five potential binding sites [37]. Interestingly, nAChRs have recently been purified from *Torpedo* electric tissue in a form that, after reconstitution, remains functionality and is amenable to high‐resolution structural analysis [45].

The postsynaptic localization of neuronal nAChRs is mediated by interactions between the intracellular domains of these receptors and cytoplasmic scaffolding proteins [46]. The nAChRs are assisted by protein and chemical chaperones, as well as auxiliary subunits, some of which act on many nAChRs, while others are more specific [46]. Recent studies, including determinations of the X‐ray crystal structures of the nearly intact α4β2 nAChR and of the ligand‐binding domains of the α9 and α2 subunits, have revealed many structurally and functionally important interactions [47].

Analyses based on proteomics, genetic approaches, and expression cloning have identified a bevy of proteins and metabolites that act at different steps in the biogenesis of nAChRs and are essential for receptor function [48]. Within the endoplasmic reticulum, chaperones mediate the folding and assembly of nAChR subunits, a process possibly reflected in the rapid association of calnexin, endoplasmic reticulum‐resident protein 57, and immunological heavy chain‐binding protein with newly synthesized subunits [49]. These interactions stabilize and sequester subunits during their assembly.

The chemical chaperones 4‐phenylbutyric acid and valproic acid promote extensive expression of α7 nAChR, which is useful for obtaining high level of human α7 nAChR for drug testing and characterization, as well as possibly also for increasing the expression of α7 in vivo [50]. Moreover, TMEM35a (NACHO), a novel chaperone expressed specifically by neurons for assembly of the homomeric α7 and of the heteromeric α3‐, α4‐, and α6‐containing nAChRs, was found to mediate the assembly of all major classes of pre‐ and post‐synaptic nAChRs tested [51]. NACHO‐knockout mice lacking both pre‐ and post‐synaptic nAChRs exhibit locomotor and cognitive abnormalities, underscoring the potential significance of this chaperone in connection with physiological and pathological processes involving nAChRs [51].

As indicated above, neuronal nAChRs are involved in many brain functions, including cognition, ability of learning and memory, arousal, cerebral blood flow and metabolism, neurotransmission, and sensory transduction [52]. Pharmacologically, these receptors are responsible for addiction to tobacco and are targeted by drugs designed to attenuate hypertension and dementia [48]. The nAChRs regulate the neurobiological processes of learning and memory in the hippocampus [53]. The α7 nAChR highly expressed throughout the hippocampus is more permeable to calcium than other subtypes of the receptors and is associated with a variety of neurological disorders and neurodegenerative diseases, making it of considerable interest in connection with efforts to combat these pathologies [54].

Neuronal nAChRs is involved in controlling resting membrane potential, regulating synaptic transmission and mediating fast excitatory transmission [55]. The honeybee brain contains at least two subtypes of nAChRs, that is, the α‐bungarotoxin‐sensitive receptor necessary for the formation of long‐term memory and the α‐bungarotoxin‐insensitive receptor involved in one‐trial acquisition of memory and in retrieval processes [56]. These findings suggests that multiple‐trial associative learning is mediated by activation of the α‐bungarotoxin‐sensitive nAChRs which, in turn, activates intracellular events leading to the formation of long‐term memory [55, 56].

The mice with α7 nAChR‐knockout show normal set‐shifting, but exhibit impaired learning processes (rule acquisition) in terms of multiple paradigms [57]. Hippocampal learning is thought to induce meta‐plasticity, which promotes subsequent learning [57]. The blocking of α7 nAChR selectively interfered with promotion of learning‐induced subsequent learning, which is thought to induce meta‐plasticity and thus promote subsequent learning [58]. Moreover, mice in which α7 nAChR has been knocked out demonstrate impairment in memory, while selective α7 agonists significantly improve their learning, memory, and attention [26]. The α7 nAChRs in limbic structures such as the hippocampus and amygdala have been demonstrated to play a critical role in connection with memory [26].

In addition, neuronal nAChRs establish close functional relationships, especially between dopaminergic and glutamatergic receptors, and these receptors can in turn cooperate with one another [59]. In dopaminergic neurons, the dopamine D3 receptor (D3R) and nAChR are assembled into a heteromeric complex (D3R‐nAChR) that is localized both in the soma and dendrites, which supports neuronal plasticity and survival [60]. Activation of nAChRs promotes morphological remodeling of dopaminergic neurons, a process that requires functional D3R [61].

The nAChRs and ionotropic glutamate receptors are often expressed on the same nerve endings [62] and are functionally inter‐dependent, with the former directly regulating the release of glutamate and other neurotransmitters [63]. This dynamic control of receptors for NMDA and alpha‐amino‐3‐hydroxy‐5‐methyl‐4‐isoxazole‐propionic acid (AMPA) by the cholinergic nicotinic system might represent an important presynaptic neuronal adaptation to exposure to nicotine [62]. Indeed, different nAChR subtypes have their specific localization and expression in excitatory and inhibitory neurons [64, 65]. Since nAChRs express more intensively in inhibitory than excitatory interneurons, their regulation of inhibitory networks in GABAergic neurons is generally stronger than the direct effect on hippocampal excitatory neurons [64, 65, 66]. In addition, the α7 nAChR subtype locates on GABAergic inhibitory interneurons and a subset of glutamatergic neurons in the hippocampus, while the α3β4 nAChR subtype seems to mainly express in glutaminergic neurons [66, 67]. Thus, the stimulation of acetylcholine on the α7 nAChR subtype mainly activates GABAergic interneurons, thereby inhibiting hippocampal excitatory neurons, while the activation of α3β4 nAChR subtype counteracts the activation of GABAergic interneurons, therefor directly stimulating glutaminergic neurons [66, 67].

## 4 The Influence of nAChRs on AD and Other Diseases Associated With Cognitive Impairment

Neurodegenerative disorders are primarily characterized by progressive loss of selectively susceptible populations of neurons, rather than the loss of specific groups of neurons due to metabolic or toxic disorders [3]. Cell death and shrinkage in a specific region of the brain is a fundamental characteristic of a variety of neurodegenerative diseases [4]. In the clinic, VD, schizophrenia, and diabetes mellitus are the most common non‐neurodegenerative diseases associated with cognitive impairment [68, 69, 70]. Interestingly, nicotinic drugs targeting nAChRs provide beneficial treatment for many forms of cognitive dysfunction [26]. Here, we discuss the associations between nAChRs and the major forms of cognitive impairment.

### 4.1 Changes in nAChRs Associated With AD

As revealed by extensive investigation, alteration in neuronal nAChRs plays an important role in the development and progression of AD [17, 25, 71]. Noninvasive PET allows early diagnosis of AD [72] and has revealed that changes in the cortical nAChRs in patients with mild AD are closely associated with cognitive attention [73]. For example, specific binding of the radioligand 2‐[^18^F]FA‐85380 BP(ND) to α4β2 nAChRs was reduced in patients with mild‐to‐moderate AD, apparently reflecting an early event in this pathological development [74]. Furthermore, the binding of [^11^C]‐(R)‐MeQAA BPND, an α7 nAChR radiotracer, in the temporal and prefrontal cholinergic projection regions was lower in patients with AD, and this decline was significantly correlated with impairment of memory and frontal function [75].

An early investigation found that the binding of [^3^H]nicotine and [^3^H]acetylcholine to nAChRs in the frontal cortex was clearly reduced in patients with AD [76], as was striatal binding of 5‐iodo‐3‐[2(S)‐2‐azetidinylmethoxy]pyridine, which targets predominantly the α4β2 subtype [77]. In addition, application of the nicotinic agonists [^3^H]epibatidine and [^3^H]nicotine, which bind with high affinity to subtypes of nAChR containing α4 and α3, respectively, either to members of the Swedish family carrying the 670/671 APP mutation (APPswe) or to sporadic cases of AD revealed less binding in various regions of the cortex [78]. Our own previous study demonstrated that the number of [^125^I]α‐bungarotoxin‐binding sites (i.e., α7 nAChR) in the temporal cortex of the brains of APPswe individuals was declined [79].

In addition, we have confirmed that in patients with AD the levels of α7, α4, and α3 subunit proteins are significantly reduced in the hippocampus, as well as in the frontal and temporal cortices [79, 80, 81, 82]. Moreover, this decreased expression of α3 was correlated to elevated expression of nuclear factor‐κB and chemokines [83]. No alterations in the levels of mRNAs encoding α3, α4, and α7 were observed, suggesting that the loss of high‐affinity epibatidine‐binding sites in patients with AD must occur at the translational and/or posttranslational level [84].

Patch‐clamp recordings show that in the cortices of patients with AD and healthy controls, the number and pattern of distribution of neurons expressing α4 and α7 nAChR mRNA are similar, whereas the corresponding neurons of patients contain approximately 30% less of these subunit proteins, which may be related to their cholinoceptive deficit [85]. Furthermore, we observed that in the peripheral blood of patients with AD the levels of α4 and β2 mRNA and activity of acetylcholine esterase were reduced, which may serve as supplementary indicators in connection with clinical diagnosis of AD [86].

Numerous reports have documented alterations in the levels of nAChR mRNAs and proteins in animal models and cultured cells designed to simulate AD [80, 86, 87]. Our earlier findings showed that treatment of PC12 cells with nanomolar concentrations of Aβ_1‐40_ or Aβ_25‐35_ decreases the number of binding sites for [^3^H]epibatidine and [^125^I]α‐bungarotoxin, as well as the levels of α3, α7, and β2 mRNA and protein [87]. These results indicate that the decline in the biosynthesis of nAChRs induced by Aβ may be attributable, at least in part, to perturbance of intracellular signal transduction pathways [80, 87].

Interestingly, the reductions in the number of nAChR‐binding sites and levels of subunit proteins caused by Aβ were largely prevented by pretreatment with an antioxidant, suggesting that oxidative stress produced by Aβ may be at least partially responsible for the loss of nAChRs associated with AD [88, 89]. Small interference RNAs (siRNAs) specifically targeting α3 nAChR in SH‐SY5Y cells enhance oxidative stress and reduce the level of αAPPs [90].

At the same time, we observed the declines in the levels of α3 and α4 mRNA and protein, as well as in the number of [^3^H]epibatidine‐binding sites on nAChRs in SH‐SY5Y cells or primary cultured neurons transfected with the APPswe gene, indicating that overexpression of the APPswe gene influences expression of nAChRs [91]. In addition, the same cellular inhibition of α7 nAChR expression with siRNAs enhanced the toxicity exerted by Aβ, whereas stimulation of this receptor by exposure to 3‐[2,4‐dimethoxybenzylidene] anabaseine, a selective agonist, attenuated this toxicity, indicating that α7 nAChR may provide neuroprotection by improving antioxidant defenses and inhibiting the toxicity of Aβ [92]. Furthermore, activation of α7 nAChR by PNU282987 attenuates the toxic effect of Aβ both in vivo and in vivo, while in mice carrying the APP/PSI mutation this activator reduces deposition of Aβ in the hippocampus, helps maintain normal synaptic morphology by partially reversing the increases in expression of associated proteins, activates the Ca^2+^ signaling pathway, and improves cognitive abilities [93].

More recently, the involvement of nAChRs in the pathogenesis of AD has been emphasized further by reports that both the nAChR and the muscarinic acetylcholine receptor (mAChR) are affected in patients with AD and that nAChRs and Aβ interact [94, 95, 96, 97]. A reduction in the level of α4 nAChR subunit protein in the brains of 12‐month‐old APP/PS2 mice is associated with clear cognitive impairment and a decrease in the accumulation of [^125^I]5IA (an imaging probe for α4β2 nAChR), indicating that such a decrease might serve as a biomarker for cognitive impairment associated with AD [98].

Interestingly, in addition to nAChRs expressed by neurons, these same receptors in astrocytes also appear to play an important role in the pathogenesis of AD, as well as in the initiation and potentiation of the early pathological changes caused by Aβ [71]. Previously, we observed elevated total number of astrocytes expressing the α7 nAChR subunit in the hippocampus and temporal cortex of both individuals carrying the APPswe mutation and those with sporadic AD, in which this elevation was positively correlated with the extent of neuropathological alterations, especially the numbers of neuritic plaques [79].

Furthermore, we found elevated levels of α7, α4, and β2 mRNA and protein in astrocytes exposed to as little as 0.1–100 nM Aβ_1‐42_, which might reflect defensive or compensatory mechanisms [99]. These results were confirmed in the Tg2576 murine model of AD, which overproduces and accumulates Aβ, by the demonstration that biologically relevant concentrations of Aβ_1‐42_ elicit α7 nAChR‐dependent elevations in the level of calcium in hippocampal CA1 astrocytes and induce NMDA receptor‐mediated slow inward currents in CA1 neurons [100].

Later, when we pretreated primary murine astrocytes with nicotine, aggregation of Aβ was inhibited markedly, while expression of endogenous αB‐crystallin was upregulated, in which the effects could be prevented by pretreatment with methyllycaconitine (MLA), a selective antagonist of α7 nAChR [101]. Furthermore, PNU282987, as a potent agonist of α7 nAChR, dramatically inhibited Aβ aggregation and upregulated the expressions of heat shock factor‐1, heat shock protein 70, and αB‐crystallin in primary astrocytes, and the effects were prevented by pretreatment with MLA [102].

Although nAChRs are the high‐affinity target for Aβ, there are still some conflicting results regarding the effect of Aβ on the receptors [103, 104]. Interestingly, recent studies concerning the cultured hippocampal neurons found that Aβ selectively binds to α7 and α4β2 nAChR subtypes in the hippocampus, without binding to α3β4, which inhibits the activity of both receptors in inhibitory neurons and thereafter leads to overexcitation of synaptic function of excitatory neurons [105, 106]. In addition, studies have shown that co‐activation of α7 and α4β2 has a greater neuroprotective effect on a cognitive impairment compared to a single receptor stimulation [107], which is required to reverse Aβ‐induced neurotoxicity [105, 106]. However, pharmacological inhibition of α7 or α4β2 nAChR subtype had no effect on working memory in nonhuman primates [108]. Therefore, understanding how Aβ affects the function of different nAChR subtypes in AD is challenging for the therapeutic target.

The loss of cholinergic tone and acetylcholine in the brain afflicted by AD is proposed to be responsible for the cognitive decline observed [97]. Whereas cholinesterase inhibitors such as donepezil, galantamine, or rivastigmine, together with memantine, a noncompetitive antagonist of the NMDA receptor, are presently at the forefront of clinical interventions for AD, new insights are bringing other drugs targeting nAChRs to center stage [109]. At the same time, available evidence indicates that therapies targeting a single aspect of AD do not effectively diminish long‐term progression and that drugs targeting more than one aspect of this disease may be more successful [97, 109]. Indeed, modulators of α7 nAChR, including nicotine and certain of its derivatives, may be of interest in this context, since they possess anti‐inflammatory and anti‐apoptotic properties, attenuate abnormal protein aggregation, and enhance cognition [110].

In an early study, we found that statins, both lipophilic and hydrophilic, induce high expression of α7 nAChR, decrease cholinesterase activities, and increase the level αAPPs in SH‐SY5Y cells, indicating that these drugs might be able to play an important neuroprotective role in connection with treatment of AD [111]. Our further observations indicate that lovastatin upregulates expression of α7 nAChR in primary cultured neurons and SH‐SY5Y cells overexpressing human APP670/671 through a mechanism involving activation of the mitogen‐activated protein kinases/extracellular signal‐regulated kinase (MAPK/ERK) pathway, which might diminish production of Aβ [112].

In addition, our results showed that scutellarin, a traditional Chinese herb, attenuated certain of the deleterious effects of Aβ, possibly by stimulating translation of nAChR mRNA and regulating cholinesterase activity [113]. Moreover, upon administration of resveratrol (a polyphenol compound isolated mainly from plants that stimulates sirtuin 1) to APP/PS1 mice, their spatial learning and memory improved and the production and aggregation of Aβ in the hippocampus and cerebral cortex declined, effects of which in the mechanism might be involved in that this compound stimulates the expression of α7 nAChR [114].

However, the extremely high‐affinity interaction between Aβ_42_ and α7 nAChR, as well as the fact that this receptor becomes rapidly desensitized, makes the development of an anti‐AD drug that directly targets α7 nAChR quite challenging [115]. Interestingly, evidence suggests that cotinine ([5S]‐1‐methyl‐5‐[3‐pyridyl]‐pyrrolidin‐2‐one), the main metabolite of nicotine, has similar beneficial properties against AD pathology as nicotine to improve memory, prevent memory loss, and lower Aβ burden in AD, as well as reduce cortical tau phosphorylation. but does not have the adverse side effects of nicotine [116, 117].

### 4.2 Changes in nAChRs Associated With PD

PD is characterized by relatively selective degeneration of dopaminergic neurons and in patients with this disease nAChRs protect against the progression in neurotoxicity induced by rotenone, 6‐hydroxydopamine, and 1‐methyl‐4‐phenyl‐1,2,3,6‐tetrahydropyridine (MPTP) [69]. A combination of immunohistochemical and stereological approaches revealed that the number of neurons expressing the α7 subunit protein was decreased in the brains of patients with PD [118]. Significant reduction in α4 and α7 nAChR subunits occurred in the cortices of patients with PD [119]. However, this reduction cannot be attributed to alterations at the transcriptional level since the number of neurons expressing α4 mRNA in the frontal cortex of patients with PD associates with cognitive dysfunction and healthy controls did not differ [120].

Previously, we examined the different subtypes of nAChRs in the brains of patients with PD and healthy age‐matched controls employing ligand binding in combination with quantification of mRNA and protein levels [121]. In these patients, the number of binding sites for [^3^H]epibatidine and level of α3 mRNA in the caudate nucleus and temporal cortex, but not in the hippocampus, were reduced; the levels of α3 subunit protein, but not of α4, attenuated in all of these regions of the brain; the levels of β2 subunit protein and mRNA lowered in the temporal cortex and hippocampus; and the levels of α7 subunit protein, but not of the corresponding mRNA, and of [^125^I]α‐bungarotoxin‐binding sites clearly elevated in the temporal cortex [121]. These findings reveal selective losses of the nAChRs containing α3 and β2 with a concomitant increase in the α7 nAChR. In patients with PD and dementia associated with Lewy bodies, striatal deficiencies in the α6 and β3 subunits tended to be greater than those in α4 and β2, which differs for the relative proportions of different nAChRs associated with AD [122]. However, reported data indicate that there is no loss of α3, α4, α7, and β2 immunoreactivity in the putamen in PD, despite a highly significant reduction in [^3^H]nicotine binding [123]. In the squirrel monkeys treated with MPTP, no changes in mRNA levels of α4, α7, β2, and β4 were found in the substantia nigra, whereas the increased α6 mRNA and reduced β3, as well as the declines in [^125^I]epibatidine binding, were detected [124].

Imaging studies on patients with PD have demonstrated a loss of striatal binding sites for 5‐[^125^I]‐A‐85380, a novel nAChR ligand interacting predominantly with the α4β2 subtype, that closely parallels the loss of nigrostriatal dopaminergic markers [77]. In such patients, the level of 2‐[^18^F]FA‐85380 binding, a measure of α4β2 nAChR availability, as determined by PET is reduced in several widespread regions of the brain and this reduction in the subcortical and cortical regions is associated with the severity of mild cognitive impairment or depression [125]. PET examination at an early stage of PD using the radiotracer 5‐[^123^I]iodo‐3‐[2(S)‐2‐azetidinylmethoxy]pyridine ([^123^I]5IA) showed that the density of nAChRs was significantly higher in the putamen, insular cortex, and supplementary motor area, as well as that the duration of the disease was positively correlated with this density in the ipsilateral putamen [126].

In addition, the anti‐inflammatory and neuroprotective properties of GTS‐21, a selective agonist of α7 nAChR, have been confirmed in a murine model of PD achieved through administration of MPTP [127]. Furthermore, when injected into the brains of these mice, GTS‐21 restores locomotor activity and attenuates the death of dopaminergic neurons, while inhibiting activation of microglia and expression of pro‐inflammatory factors [127].

Notably, the mutant form of kinase 2 containing a leucine‐rich repeat, the most common genetic determinant of PD, prevents the localization of both the D3R and nAChRs to the neuronal membrane, as well as the formation of the D3R‐nAChR heteromer crucial for neuronal homeostasis and the health of dopaminergic neurons [59].

### 4.3 Changes in nAChRs Associated With VD

We have reported that the level of α4 mRNA, but not β2 in blood leukocytes from patients with VD is lowered in a manner that correlates significantly with their scores on clinical cognitive tests [128]. Moreover, the declined levels of both α4 and β2 mRNA are confirmed in the blood leukocytes and hippocampus of the brains of ischemic rats, and the attenuated levels of these subunit proteins in the hippocampus of both patients and ischemic rats are further observed [128]. Interestingly, these changes were correlated with impaired learning and memory. In rats with chronic cerebral hypoperfusion, tropisetron, an antagonist of 5‐hydroxytryptamine (5‐HT3) with antiemetic effects and also a partial agonist of α7 nAChR, attenuated neurotoxicity, including the impairment in working and reference memory, the increase in serum levels of interleukin‐6, and the reductions in the number of CA1 neurons and in expression of the serotonin‐reuptake transporter [129].

In addition, in patients with VD the reduced uptake of [^123^I]‐5IA‐85,380 was limited to subcortical regions of the brain and the normalized elevation of [^123^I]‐5IA‐85,380 uptake in the cuneus might be a compensatory response to the reduced cholinergic activity in the dorsal thalamus [130]. Giving nicotine to ischemic rats showed better memory and the increased mRNAs of α4 and β2 nAChR [131]. The auricular electrical stimulation at 15 Hz rapidly elevated cortical blood flow in the middle cerebral artery and increased the numbers of cells immunostaining for α4 nAChR subunit, along with the overall expression of this protein [132].

However, the brains of VD patients examined postmortem exhibited no loss of 5‐[^125^I]‐A‐85380 binding to α4β2 nAChR [77], and neither their number of epibatidine‐ and α‐bungarotoxin‐binding sites nor immunostaining for α4 and α7 altered [133]. Although the polymorphism in α7 nAChR may be involved in the development of AD, Lewy bodies, and Pick's disease, it is unlikely to play an important role in the pathogenesis of VD [134].

### 4.4 Changes in nAChRs Associated With Schizophrenia

Schizophrenia induces not only devastating hallucinations and delusions, but cognitive impairment as well, including the inability to focus one's attention [70]. Extensive evidence indicates that nAChRs play a role in connection with the cognitive symptoms associated with schizophrenia and α7 nAChR has been proposed to be a potentially valuable target for the development of effective therapeutic drugs [135].

In an early study, we observed a lowered level of α7 subunit protein in the frontal, but not the parietal cortex of schizophrenics [82]. In fact, the gene encoding α7 nAChR, located on chromosome 15q14, is related to the heritability of both schizophrenia and bipolar affective disorder, and in particular, the heritability of a deficit in inhibitory neuronal function is associated with these illnesses, which of the conclusion has received further support from psychophysiological and genetic investigations [136]. Deficits in auditory P50 that evoke potential suppression of schizophrenic symptoms are associated with reduced density of α7 nAChR in the brain [137]. Some agonists (e.g., DMXB‐A and tropisetron) at α7 nAChR can improve P50 deficits in patients with schizophrenia [137]. The level of α7 nAChR mRNA in the peripheral blood lymphocytes of schizophrenic patients is lowered, independently of whether they are smokers or not [138]. Indeed, most individuals suffering from schizophrenia are heavy smokers [139] and despite the negative health consequences, it has been proposed that smoking may attenuate the symptoms associated with this disorder [140].

In addition to the involvement of the CHRNA7 polymorphism in nAChR genes, in particular the D15S1360 associated with smoking by individuals with schizophrenia, there appears to be a significant association between the CHRNA4 rs3746372 allele 1 and smoking with a large number of cigarettes daily [141]. Although nicotine can temporarily reverse the diminished auditory sensory gating experienced by individuals with schizophrenia, this effect is lost upon chronic exposure to nicotine due to desensitization of nAChRs [70]. Nicotine upregulates expression of α4β2 nAChR, and it is possible that the beneficial effects of nicotine described by patients with schizophrenia may be partly due to this compensation for their loss of α4β2 nAChR [142]. In addition, in the cingulate cortex of these patients opposite changes, that is, an increase in α4β2 and decrease in α7 nAChRs, were observed, perhaps reflecting the involvement of two different nAChR‐dependent mechanisms in schizophrenia [143].

During the past 25 years, extensive preclinical and some early clinical evidence has suggested that ligands for nAChRs might have therapeutic value in connection with neurological and psychiatric disorders [144]. However, up to now there are still many limitations to the application of nAChR drugs, such as the translation of preclinical models to human disease practice and pharmacological effects of receptors involving agonists, antagonists, or receptor desensitization, which have not been addressed [144].

### 4.5 Changes in nAChRs Associated With Diabetes Mellitus

Recently, clinical guidelines concerning diabetes mellitus have begun to emphasize the importance of managing the associated cognitive impairment [68]. This impairment occurs in different stages, each with its own features, age‐dependency, prognosis, and, most likely, underlying mechanism [145].

In this context, studies on nAChRs have mostly involved T2DM, although type 1 diabetes mellitus also causes brain damage [146]. Dysregulation of metabolism and immune function associated with obesity are also associated with chronic inflammation, a critical step in the pathogenesis of insulin resistance and T2DM [147]. The cholinergic anti‐inflammatory reflex (CAIR) has been implicated in attenuating inflammation and metabolic complications related to obesity [147]. The α7 nAChR appears to play an important role in the control of such inflammation [148]. Vagovagal reflexes are an integral component of the CAIR, whose anti‐inflammatory effects are mediated by the action of acetylcholine acting at α7 nAChR located on cells of the immune system [148]. By increasing cytosolic levels of Ca^2+^, GTS‐21 enhances secretion of glucagon‐like peptide‐1 (GLP‐1), while also unveiling Ca^2+^‐ and phosphoinositide 3‐kinase (PI_3_K)‐dependent activation of α7 nAChR that promotes the survival of L cells [149]. In fact, in db/db (diabetes animal model) mice, a single or multiple doses of GTS‐21 lower levels of blood glucose in an oral glucose tolerance test with a dose‐dependent manner [150]. This effect could be reproduced by PNU282987 for upregulating α7 nAChR, suggesting that such agonist improves soral glucose tolerance, at least in diabetic mice [150].

In addition, oral administration of TC‐7020 to db/db mice reduced their food intake and weight gain, attenuated their elevated blood levels of glucose and glycated hemoglobin, and lowered their elevated plasma levels of triglycerides and tumor necrosis factor (TNF)‐α [151]. These changes were reversed by the α7 nAChR‐selective antagonist MLA, strongly indicating the involvement of this receptor. In a recent investigation in our laboratory, we found that the levels of α7 and α4 subunit proteins were lowered and the rate of apoptosis among neurons elevated in the (postmortem) hippocampus of patients with diabetes mellitus and db/db mice [152]. Furthermore, the db/db mice exhibited the impaired cognition, as well as an elevated level of pro‐apoptotic protein and the reduced levels of anti‐apoptotic and synaptic proteins in the brain [152]. These changes in nAChR subunits and apoptosis might help explain the impaired cognition associated with T2DM.

### 4.6 Therapeutic Potential for AD and Other Cognitive Diseases by Employing nAChR Ligands

A large number of experimental results suggest that nAChR ligands may be a potential therapeutic agent for some neurodegenerative or neuropsychiatric diseases. However, nAChR ligands have not been successful in clinical trials for AD and schizophrenia yet, and few nAChR ligands approved for the treatment of any clinical condition [153].

The tobacco alkaloid nicotine was first shown to improve cognitive function in nicotine deprived smokers, nonsmokers, and laboratory animals [154], including sustained attention and distraction, working memory, recognition memory, and executive function [155]. Cytisine, a plant‐based partial agonist of nAChRs, has been used to treat tobacco dependence for decades, which is available as a generic or prescription medication in many countries [156]. OC‐01 (varenicline solution), a nAChR agonist nasal spray, resulted in significant improvements in signs and symptoms of dry eye disease, was well tolerated, and warranted for further clinical investigation [157].

Interestingly, varenicline, a α4β2 nAChR partial agonist, was tolerated well, enhanced attention, and altered gait performance of PD patients, in which the results are consistent with target engagement [158]. However, it has been reported that most of identified clinical trials for nAChR ligands were phase II trials, with some of them classified as ongoing for several years [159]. Even though most of the physical side effects of cholinergic agonists were reported to be well tolerated and some trials with improvements in attention, the efficacy of these drugs in other cognitive and behavioral outcomes remains highly controversial.

Importantly, since the attenuated acetylcholine signaling and significant reduction in the expression of nAChRs in the brains of AD patients and other dementias have been reported in several molecular biological and in situ labeling studies, the modulation of the functional deficit of the cholinergic system as a pharmacological target could therefore have a clinical benefit, which is not to be neglected [159]. In the mechanisms connecting ligand binding to channel activation in nAChRs for which structures of extracellular and transmembrane domains are available more understanding is needed [160]. By revealing how specific ligand regulates the different conformational states of nAChRs as its own characteristic function, it is expected to further develop new nicotine receptor ligand drugs for the treatment of cognitive impairment.

## 5 Conclusions

The many different types of cognitive disturbance involve highly complex and differing pathogeneses. Although the structure, metabolism, and function of neuronal nAChRs in the human brain are well understood, the role of these receptors in the development of cognitive disorders has not yet been extensively explored. Nevertheless, it is already evident that the expressions of different nAChRs in different cognitive disorders are quite dissimilar, indicating the involvement of nAChRs in a variety of underlying mechanisms.

Clearly, nAChRs play an important role in the pathology of AD, the most common neurodegenerative disease. A lot of studies report that in association with the development and progression of AD, the binding sites on several subtypes of nAChRs (mainly α4β2 and α7) in the brain are reduced in number, which reflects lowered expression of the subunit proteins. Specially, expressions of α7 and α4β2 nAChRs in astrocytes often demonstrate a compensatory increase and overexpression of astrocytic α7 nAChR might serve as an early marker of reactive astrogliosis in patients with AD. In the case of PD, the changes in nAChRs reported were inconsistent, but manifested primarily as decreased expression of α3 mRNA and protein, as well as attenuated density of the receptors containing these subunits in the brain; increased compensatory expression of α7; an unchanged or lowered level of α4β2; and raised α6 and reduced β3, both are uncommon types of nAChRs. In patients with VD and ischemic rats, the levels of α4β2 nAChR in peripheral blood and brain tissues were lowered, but the changes in other receptor subunits were less clear. In the case of schizophrenia, the brain level of α7 nAChR declines, while that of α4β2 is elevated, but the underlying mechanism(s) remains far from clear. At the onset of diabetes mellitus, expression of both α7 and α4β2 nAChRs is reduced. The changes in nAChRs in these types of cognitive impairment above are summered in Table 1.

**TABLE 1:** Changes in nAChRs in different type of cognitive impairment.

Types	Contents	Main acting characteristics	Main references
AD	PET	Reduced α4β2 and α7 in human brain	Kadir et al., 2006 [73]; Kendziorra et al., 2011 [74]; Nakaizumi et al., 2018 [75]; Nordberg and Winblad, 1986 [76]; Marutle et al., 1999 [78]; Yu et al., 2005 [79]; Ren et al., 2020 [80]; Zhang et al., 2010 [86]; Guan et al., 2001 [87]; Qi et al., 2005 [88]; Guan et al., 2003 [89]; Tang et al., 2008 [90]; An et al., 2010 [91]; Qi et al., 2007 [92]; Wang et al., 2020 [93]; Matsuura et al., 2019 [98]; Xiu et al., 2005 [99]; Pirttimaki et al., 2013 [100]; Ren et al., 2019 [101]; Echeverria et al., 2016 [110]; Roensch et al., 2007 [111]; Zhao et al., 2018 [112]; Guo et al., 2011 [113]; Cao et al., 2020 [114]
	Binding sites	Reduced binding sites for α4, α7, and α3 in human and animal brains and cultured cells exposed with Aβ	
	Protein and mRNA	Reduced α4, β2, α7, α3 proteins and α4, β2 mRNAs in human blood; increased α7, α4, β2 proteins in astrocytes	
	Agonists	Activated α7 and α4	
PD	PET	Reduced α4β2 but increased α7 in human brains	Pimlott et al., 2004 [77]; Banerjee et al., 2000 [118]; Burghaus et al., 2003 [119]; Guan et al., 2002 [121]; Gotti et al., 2006 [122]; Quik et al., 2000 [124]; Isaias et al., 2014 [126]; Park et al., 2022 [127]
	Binding sites	Reduced binding sites for α3, α4, but increased α7 in human brain	
	Protein and mRNA level	Reduced protein levels of α3, α6, β2, β3, but increased α7 in human brains Reduced mRNA levels of α3, β2, β3, but increased α6 in human or animal brains	
	Agonists	Activated α7	
VD	Protein and mRNA level	Reduced protein levels of α4, β2, β3, but increased α7 in human and animal brains Reduced mRNA levels of α4, β2, β3 mRNA in human brain or blood	Xiao et al., 2016 [128]; Divanbeigi, et al., 2020 [129]; Colloby et al., 2011; Han et al., 2020 [131]; Huang et al., 2019 [132]; Martin‐Ruiz et al., 2000 [133]
	Agonists	Activated α7 and α4	
Sch	Binding sites	Reduced binding sites for α7 and increased for α4 in human brain	Martin and Freedman, 2007 [70]; Guan et al., 1999 [82]; Freedman et al., 2000 [136]; Ishikawa and Hashimoto, 2011 [137]; Perl et al., 2003 [138]; Durany et al., 2000; Marutle et al., 2001 [143]
	Protein and mRNA level	Reduced protein levels of α7 in human brains Reduced mRNA levels of α7 in human brain or blood	
	Agonists	Activated α7 and α4	
DM	Binding sites	Reduced or increased binding sites for α4 and α7 in human brain	Wang et al., 2018 [149]; Meng et al., 2022 [150]; Marrero et al., 2010 [151]; Xu et al., 2020 [152]
	Protein and mRNA level	Reduced protein levels of α7 and α4, β2 in human and animal brains	
	Agonists	Activated α7	

A variety of nAChR agonists and inhibitors have been and are being tested for treatment of many different types of cognitive impairment, which is a promising class of drugs that offers good prospects for improving cognitive deficit. In particular, the beneficial effects of α7 nAChR agonists for patients with AD and schizophrenia are now fully recognized. However, future choices of the appropriate nAChR subtype(s) or subunit(s) as the therapeutic target for each particular form of cognitive impairment will require a much more detailed understanding of the nature of the individual diseases and their underlying mechanisms, especially with respect to the involvement of nAChRs.

## Author Contributions

**Zhi‐Zhong Guan:** Material preparation, data collection, analysis, and writing for the first and revised manuscript.

## Conflicts of Interest

The author declares no conflicts of interest.

## Data Availability

The data that support the findings of this study are available on request from the corresponding author.
